# Supplementary material for: A Humanized Yeast Phenomic Model of Deoxycytidine Kinase to Predict Genetic Buffering of Nucleoside Analog Cytotoxicity
Source: Genes (Basel). 2019 Sep 30;10(10):770. doi: 10.3390/genes10100770 (PMC6826991; doi:10.3390/genes10100770)
Supplement: Supplementary file 1 [file genes-10-00770-s001.zip › Additional_Files/Additional_File7_GOTermHeatmaps.zip!/Additional_File7_GOTermHeatmaps/transcription_factor_binding.pdf]

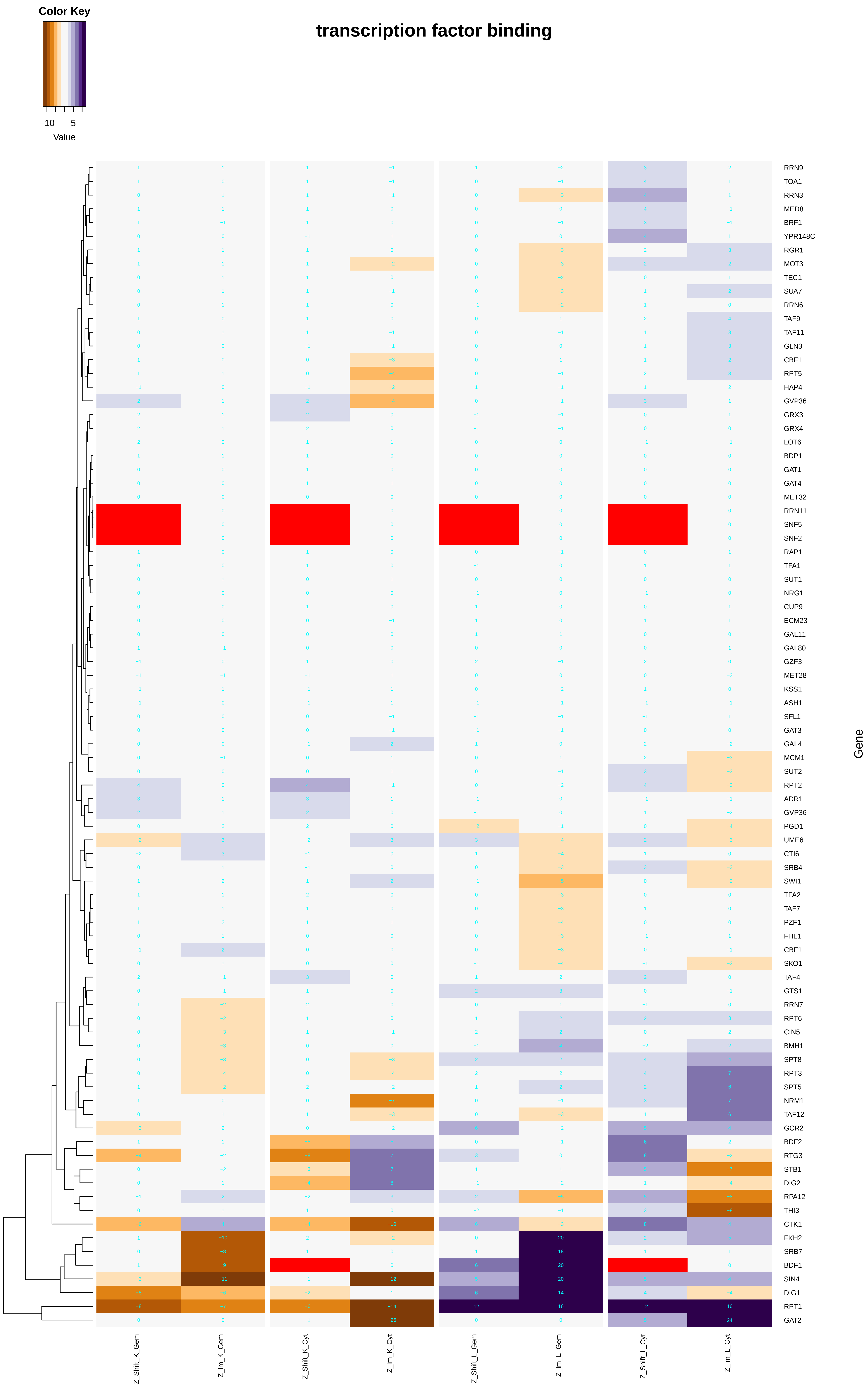
Color Key
−10
5
Value
transcription factor binding
RRN9
TOA1
RRN3
MED8
BRF1
YPR148C
RGR1
MOT3
TEC1
SUA7
RRN6
TAF9
TAF11
GLN3
CBF1
RPT5
HAP4
GVP36
GRX3
GRX4
LOT6
BDP1
GAT1
GAT4
MET32
RRN11
SNF5
SNF2
RAP1
TFA1
SUT1
NRG1
CUP9
ECM23
GAL11
GAL80
GZF3
MET28
KSS1
ASH1
SFL1
GAT3
GAL4
MCM1
SUT2
RPT2
ADR1
GVP36
PGD1
UME6
CTI6
SRB4
SWI1
TFA2
TAF7
PZF1
FHL1
CBF1
SKO1
TAF4
GTS1
RRN7
RPT6
CIN5
BMH1
SPT8
RPT3
SPT5
NRM1
TAF12
GCR2
BDF2
RTG3
STB1
DIG2
RPA12
THI3
CTK1
FKH2
SRB7
BDF1
SIN4
DIG1
RPT1
GAT2
Gene
Z_Shift_K_Gem
Z_Im_K_Gem
Z_Shift_K_Cyt
Z_Im_K_Cyt
Z_Shift_L_Gem
Z_Im_L_Gem
Z_Shift_L_Cyt
Z_Im_L_Cyt

RNA polymerase III transcription factor binding

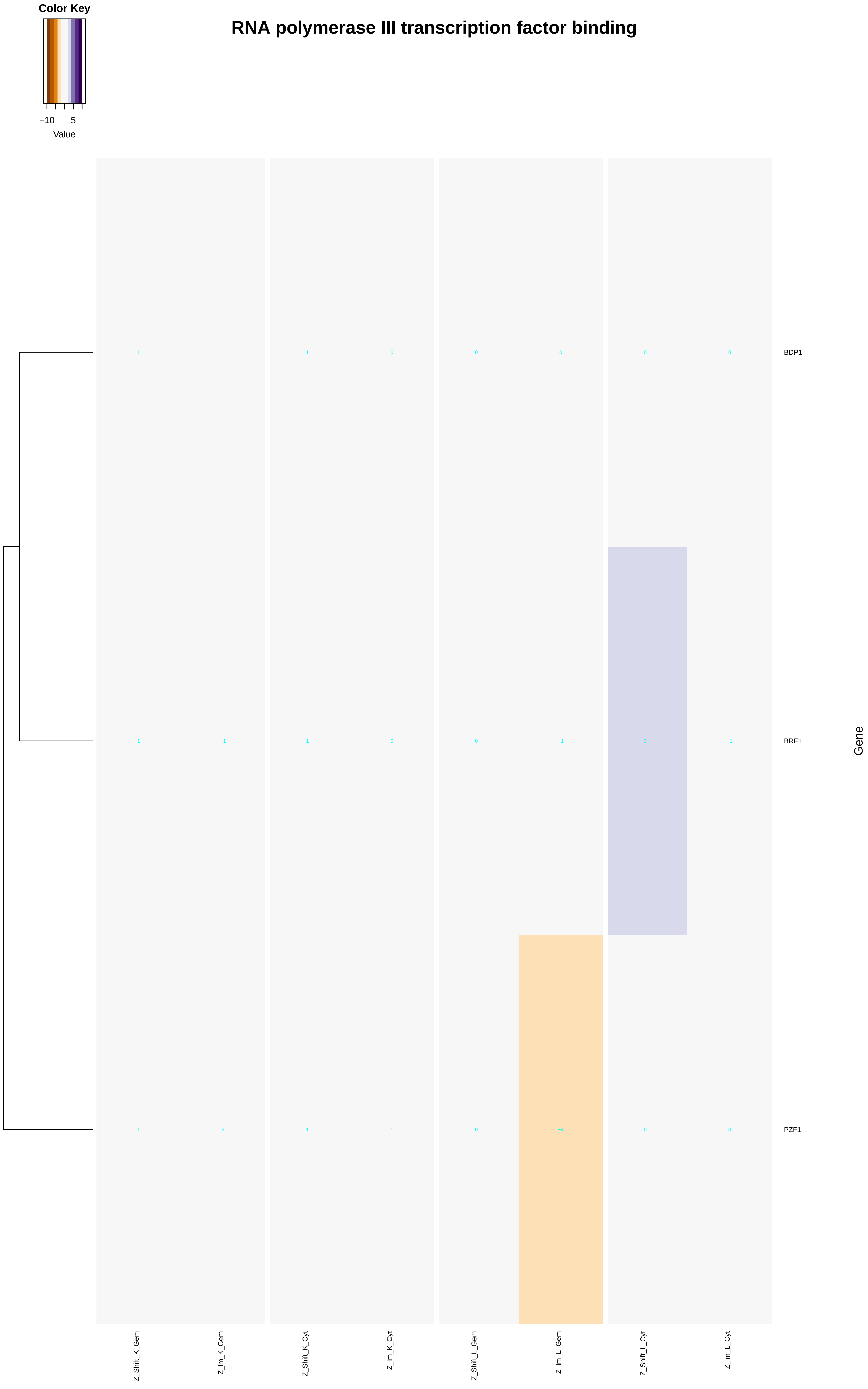

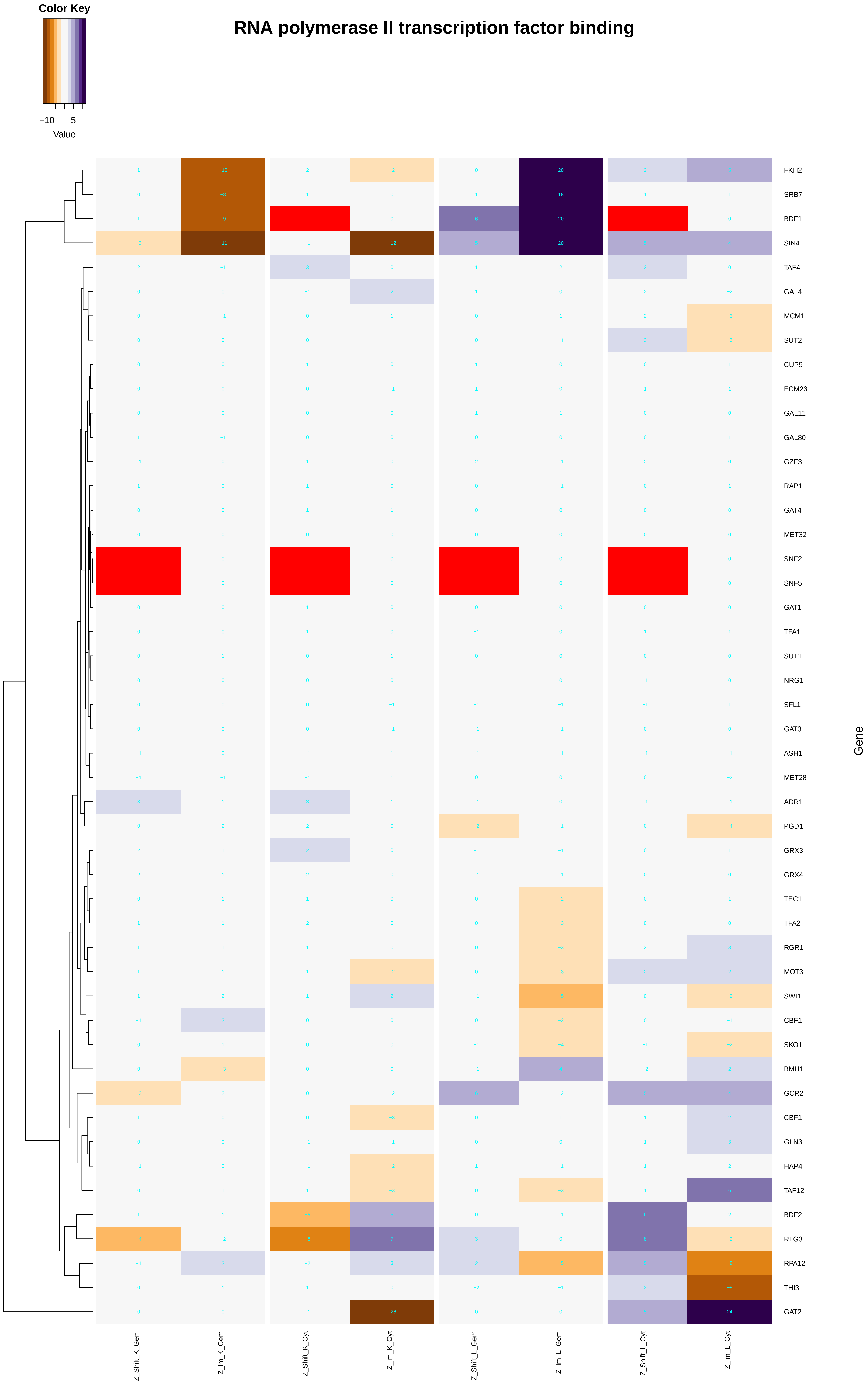
Color Key
−10
5
Value
RNA polymerase II transcription factor binding
FKH2
SRB7
BDF1
SIN4
TAF4
GAL4
MCM1
SUT2
CUP9
ECM23
GAL11
GAL80
GZF3
RAP1
GAT4
MET32
SNF2
SNF5
GAT1
TFA1
SUT1
NRG1
SFL1
GAT3
ASH1
MET28
ADR1
PGD1
GRX3
GRX4
TEC1
TFA2
RGR1
MOT3
SWI1
CBF1
SKO1
BMH1
GCR2
CBF1
GLN3
HAP4
TAF12
BDF2
RTG3
RPA12
THI3
GAT2
Gene
Z_Shift_K_Gem
Z_Im_K_Gem
Z_Shift_K_Cyt
Z_Im_K_Cyt
Z_Shift_L_Gem
Z_Im_L_Gem
Z_Shift_L_Cyt
Z_Im_L_Cyt

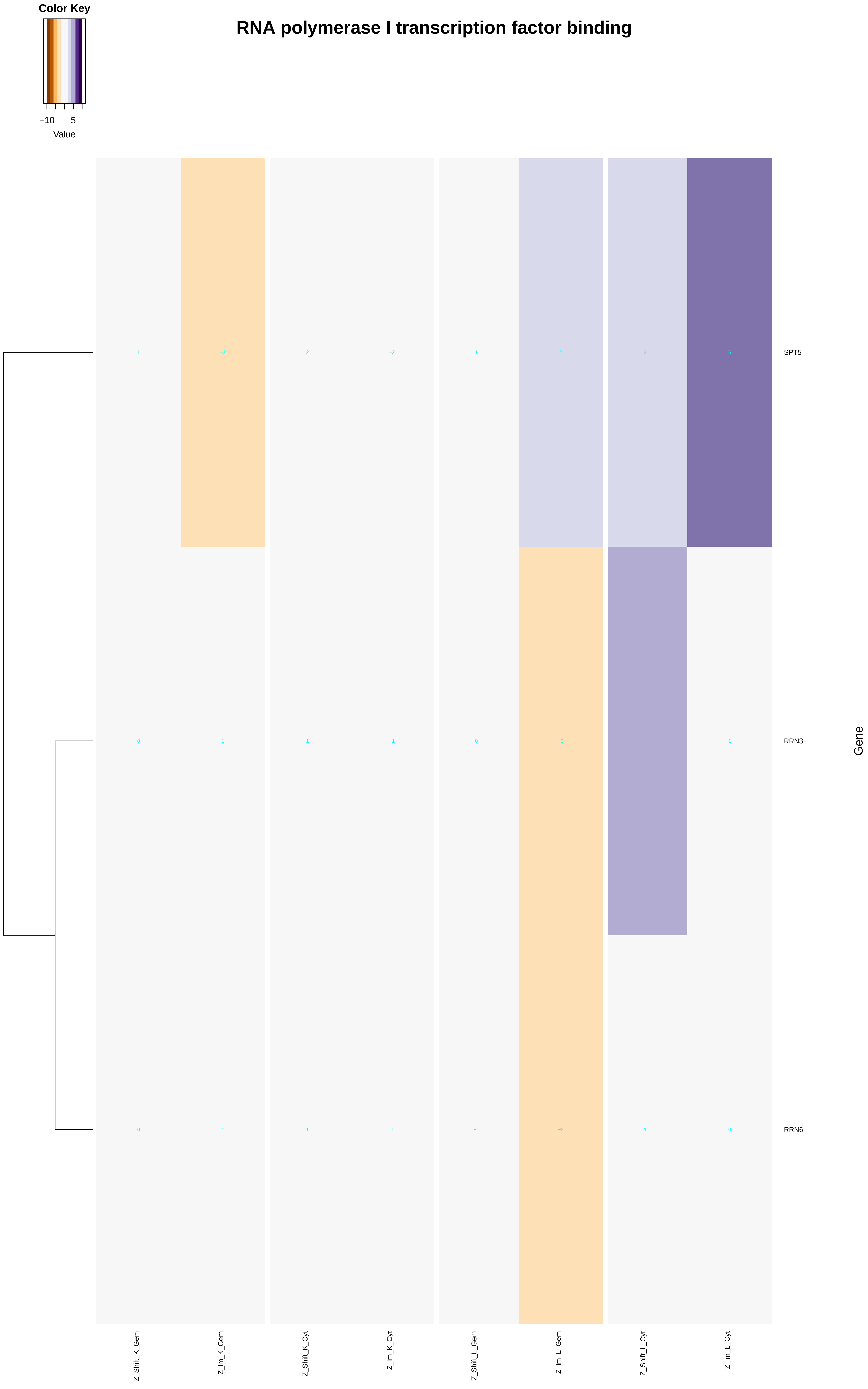

Color Key
−10
5
Value
RNA polymerase I transcription factor binding
1
−2
2
−2
1
2
2
6
SPT5
0
1
1
−1
0
−3
4
1
RRN3
0
1
1
0
−1
−2
1
0
RRN6
Gene
Z_Shift_K_Gem
Z_Im_K_Gem
Z_Shift_K_Cyt
Z_Im_K_Cyt
Z_Shift_L_Gem
Z_Im_L_Gem
Z_Shift_L_Cyt
Z_Im_L_Cyt

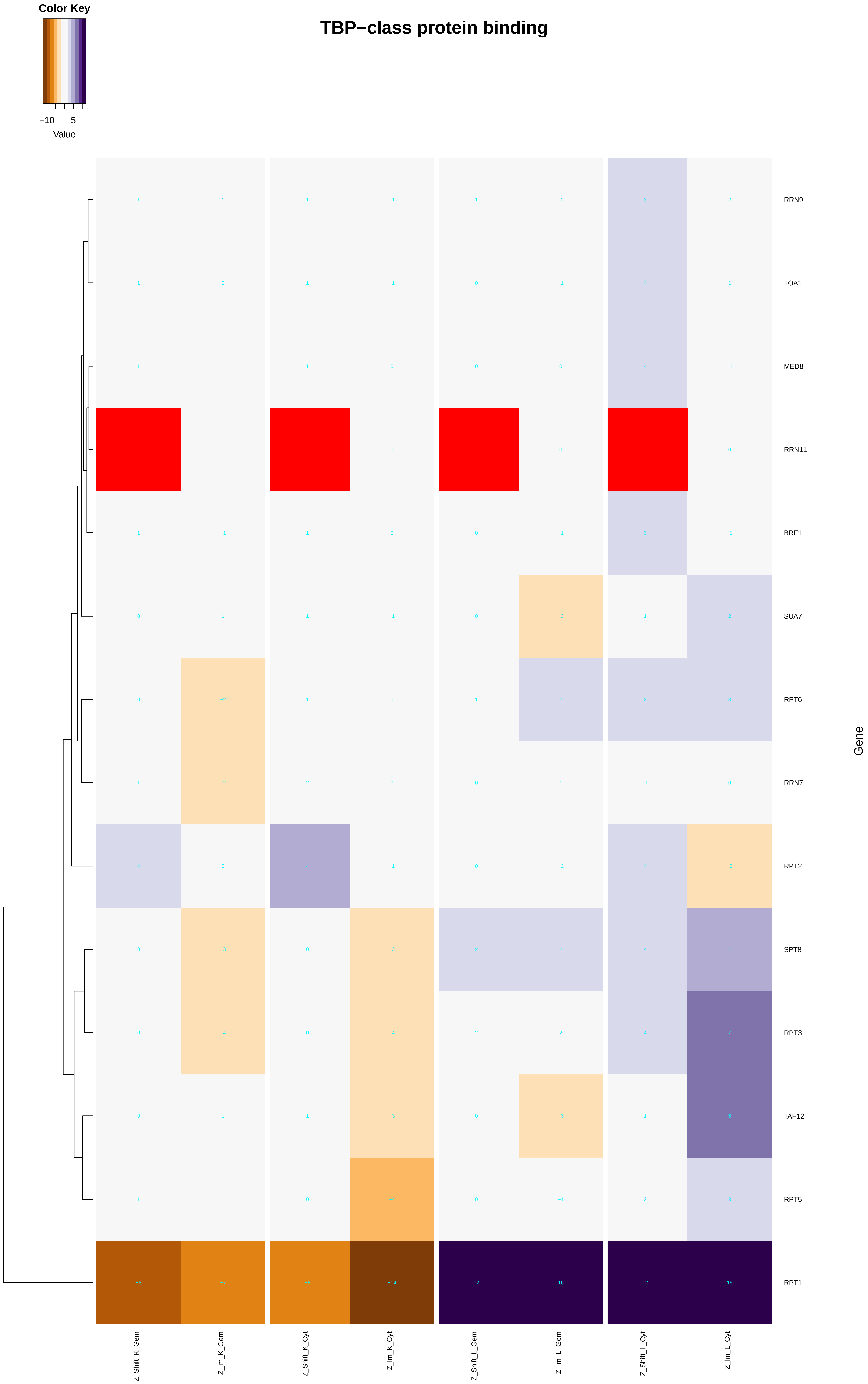
Color Key
−10
5
Value
TBP−class protein binding
RRN9
TOA1
MED8
RRN11
BRF1
SUA7
RPT6
RRN7
RPT2
SPT8
RPT3
TAF12
RPT5
RPT1
Gene
Z_Shift_K_Gem
Z_Im_K_Gem
Z_Shift_K_Cyt
Z_Im_K_Cyt
Z_Shift_L_Gem
Z_Im_L_Gem
Z_Shift_L_Cyt
Z_Im_L_Cyt

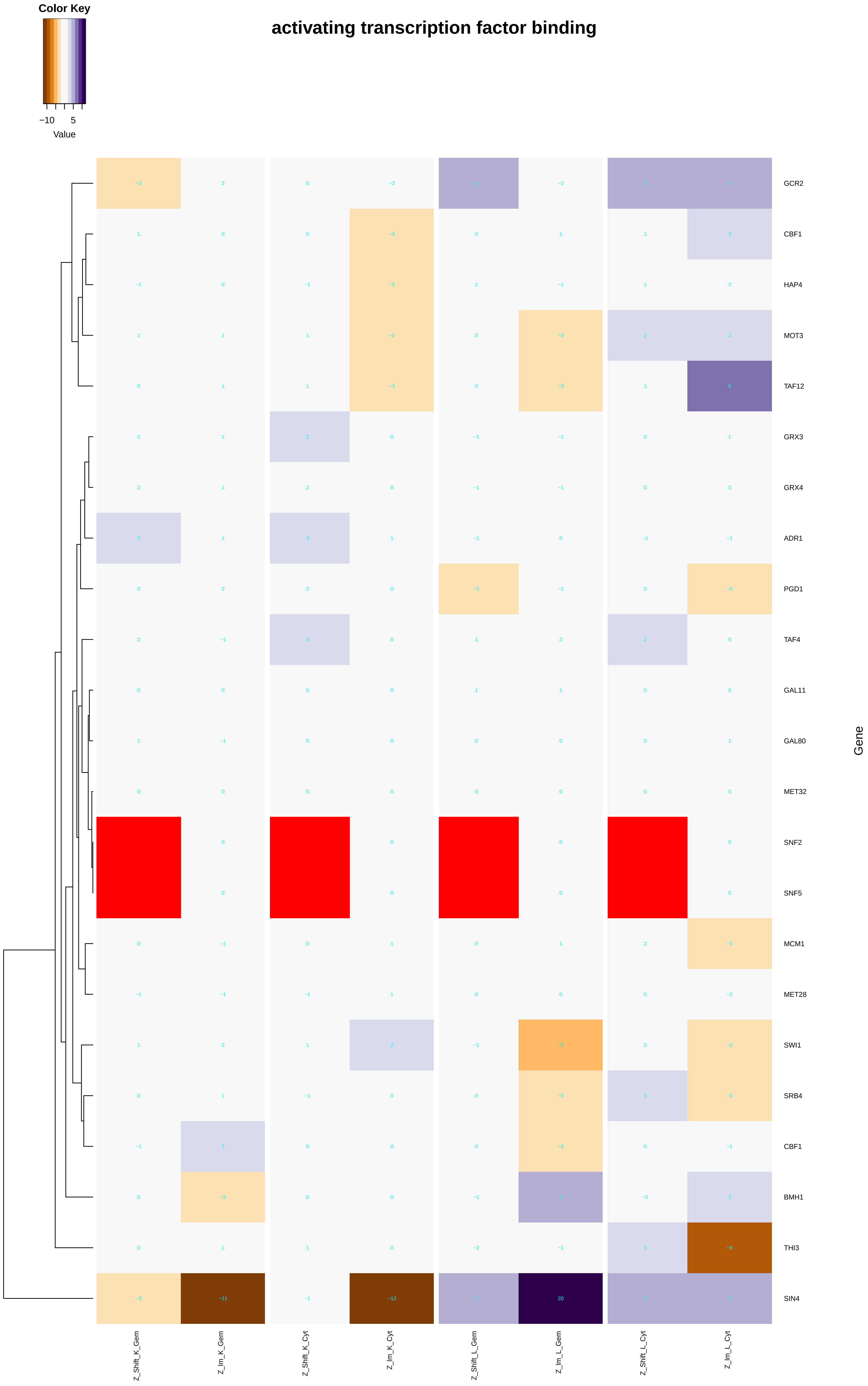
activating transcription factor binding
Color Key
−10
5
Value
GCR2
CBF1
HAP4
MOT3
TAF12
GRX3
GRX4
ADR1
PGD1
TAF4
GAL11
GAL80
MET32
SNF2
SNF5
MCM1
MET28
SWI1
SRB4
CBF1
BMH1
THI3
SIN4
Gene
Z_Shift_K_Gem
Z_Im_K_Gem
Z_Shift_K_Cyt
Z_Im_K_Cyt
Z_Shift_L_Gem
Z_Im_L_Gem
Z_Shift_L_Cyt
Z_Im_L_Cyt

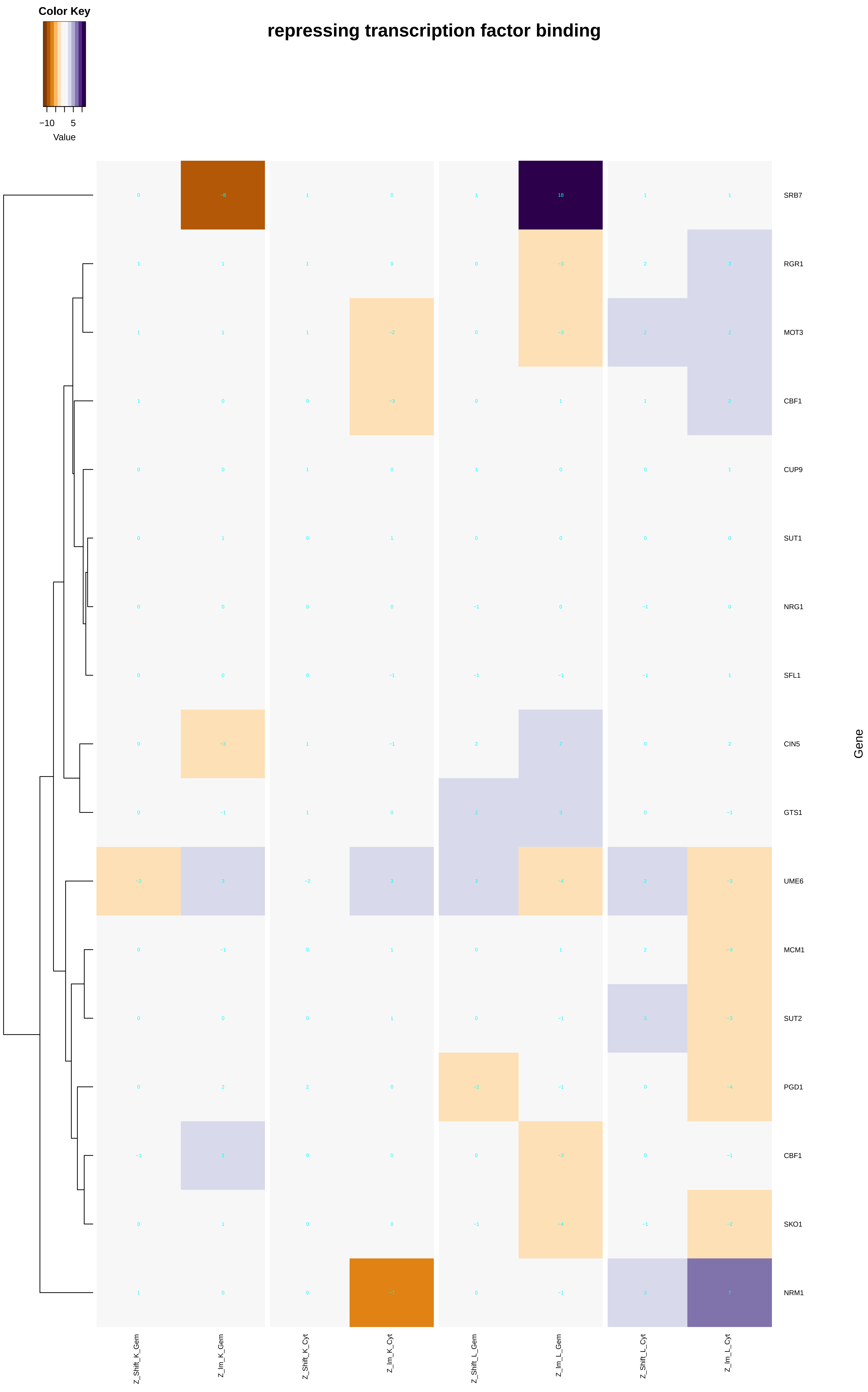
Color Key
−10 5
Value
repressing transcription factor binding
SRB7
RGR1
MOT3
CBF1
CUP9
SUT1
NRG1
SFL1
CIN5
GTS1
UME6
MCM1
SUT2
PGD1
CBF1
SKO1
NRM1
Gene
Z_Shift_K_Gem
Z_Im_K_Gem
Z_Shift_K_Cyt
Z_Im_K_Cyt
Z_Shift_L_Gem
Z_Im_L_Gem
Z_Shift_L_Cyt
Z_Im_L_Cyt

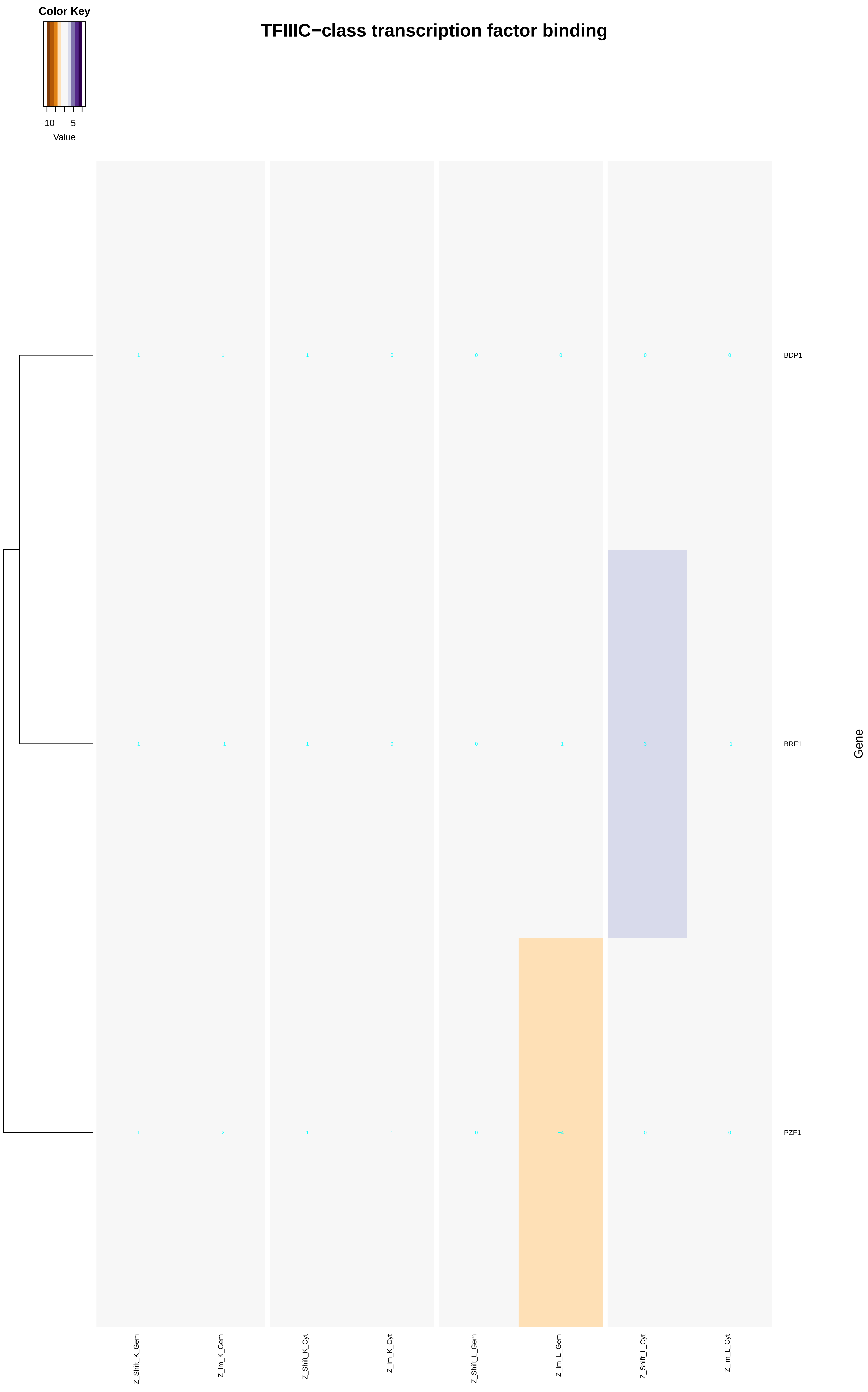

Color Key
−10
5
Value
TFIIIC−class transcription factor binding
BDP1
BRF1
PZF1
Gene
1
1
1
0
0
0
0
0
1
−1
1
0
0
−1
3
−1
1
2
1
1
0
−4
0
0
Z_Shift_K_Gem
Z_Im_K_Gem
Z_Shift_K_Cyt
Z_Im_K_Cyt
Z_Shift_L_Gem
Z_Im_L_Gem
Z_Shift_L_Cyt
Z_Im_L_Cyt

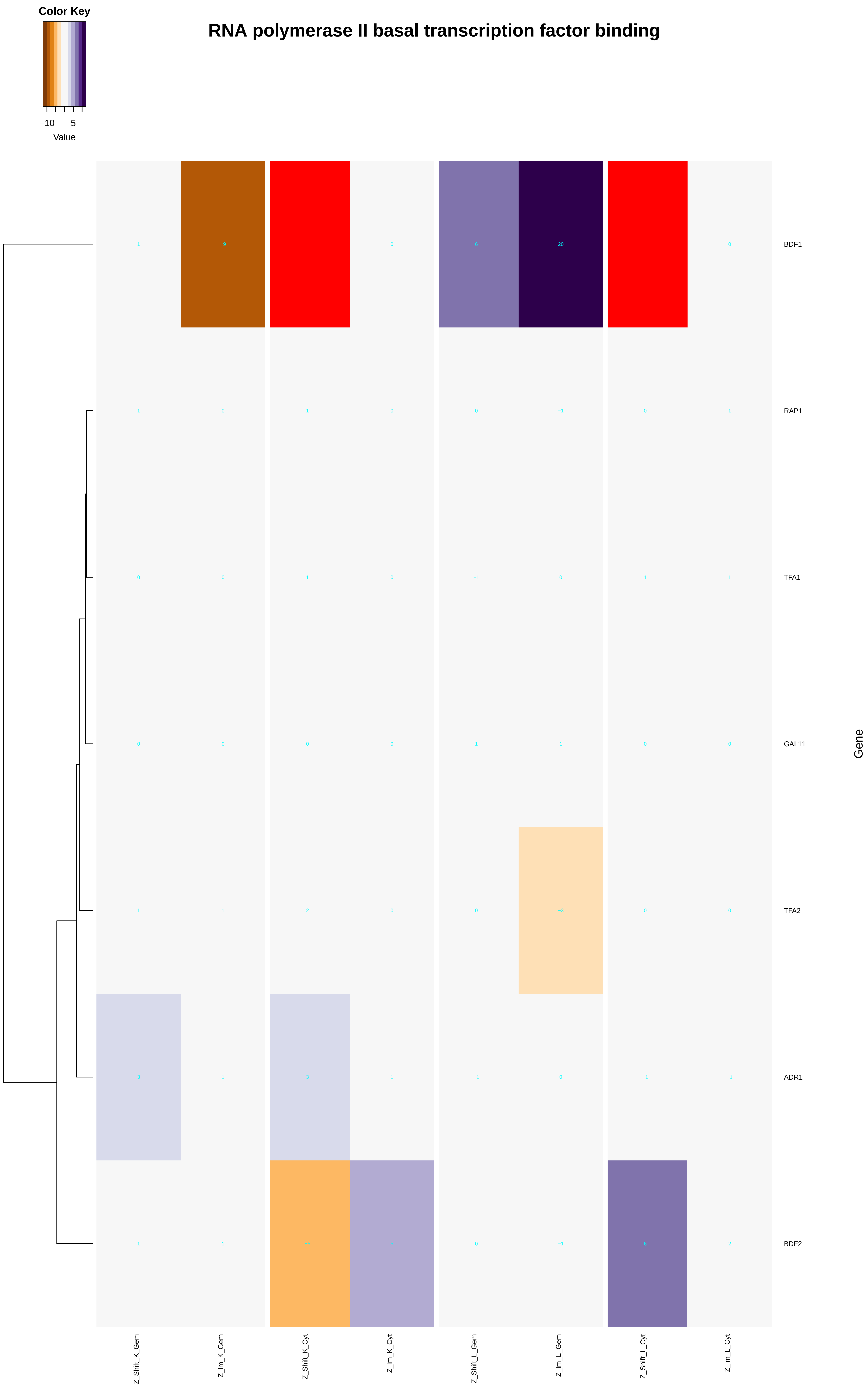

Color Key
−10
5
Value
RNA polymerase II basal transcription factor binding
BDF1
RAP1
TFA1
GAL11
TFA2
ADR1
BDF2
Gene
Z_Shift_K_Gem
Z_Im_K_Gem
Z_Shift_K_Cyt
Z_Im_K_Cyt
Z_Shift_L_Gem
Z_Im_L_Gem
Z_Shift_L_Cyt
Z_Im_L_Cyt

RNA polymerase II activating transcription factor binding

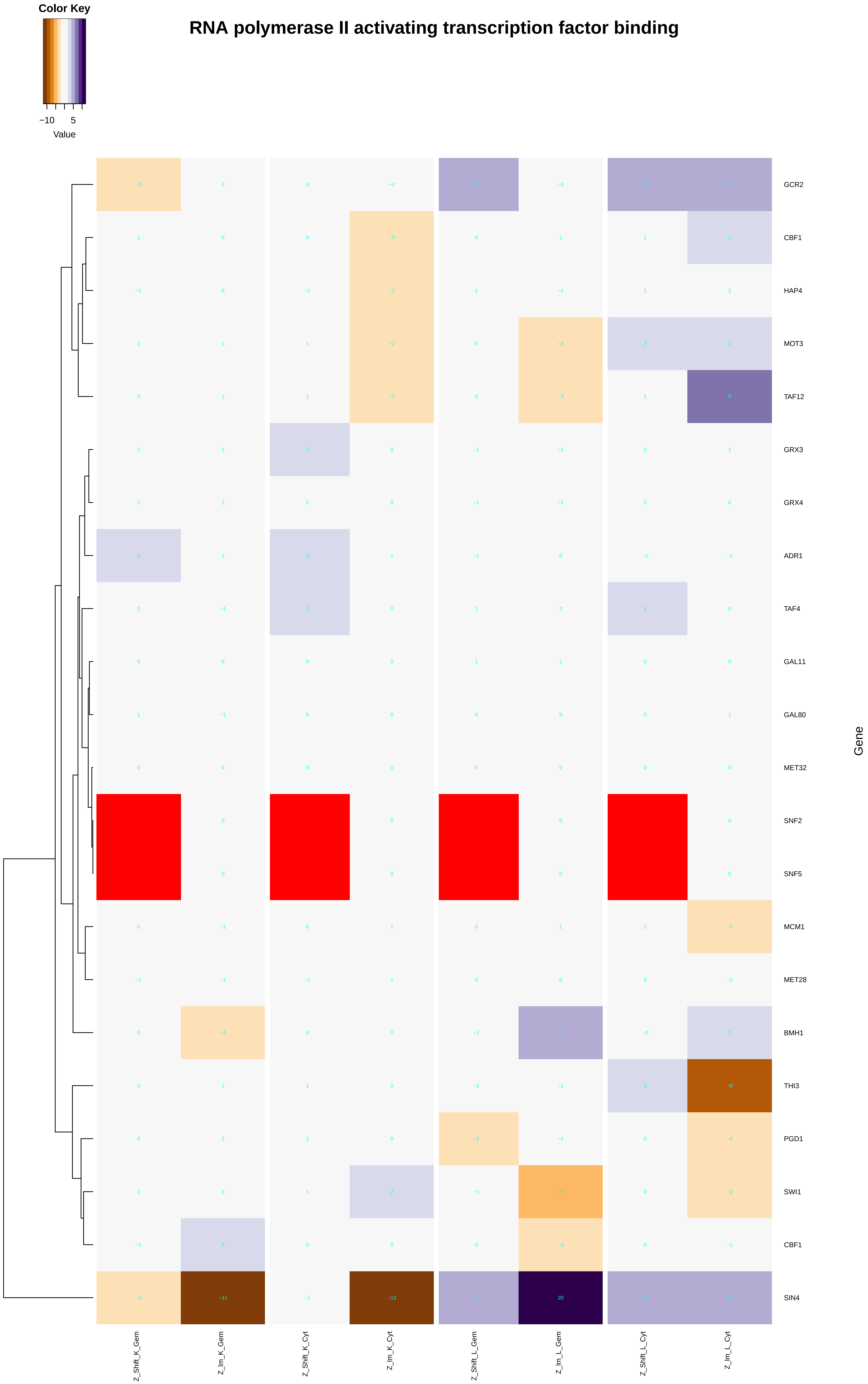

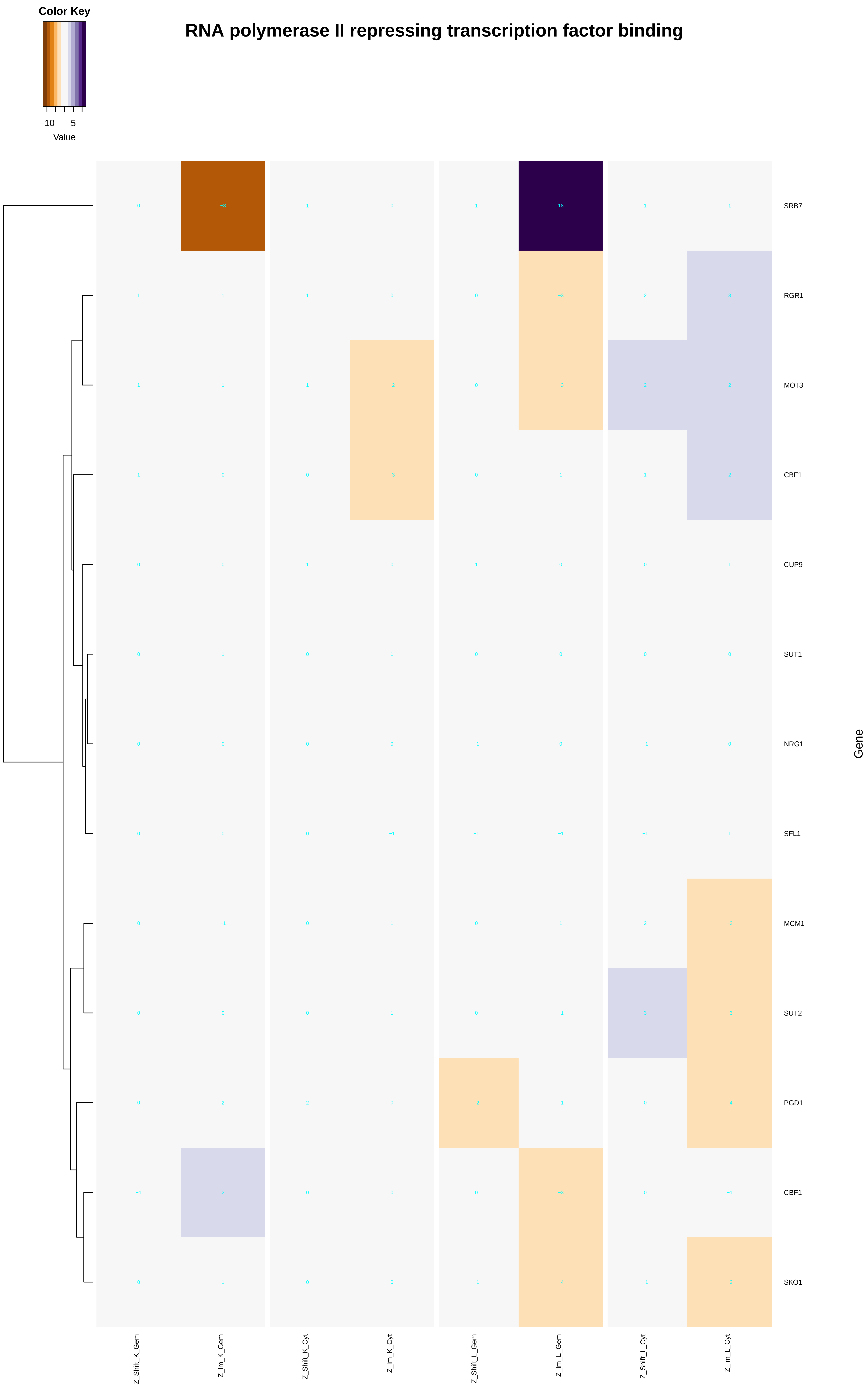
RNA polymerase II repressing transcription factor binding
Color Key
−10
5
Value
SRB7
RGR1
MOT3
CBF1
CUP9
SUT1
NRG1
SFL1
MCM1
SUT2
PGD1
CBF1
SKO1
Gene
Z_Shift_K_Gem
Z_Im_K_Gem
Z_Shift_K_Cyt
Z_Im_K_Cyt
Z_Shift_L_Gem
Z_Im_L_Gem
Z_Shift_L_Cyt
Z_Im_L_Cyt

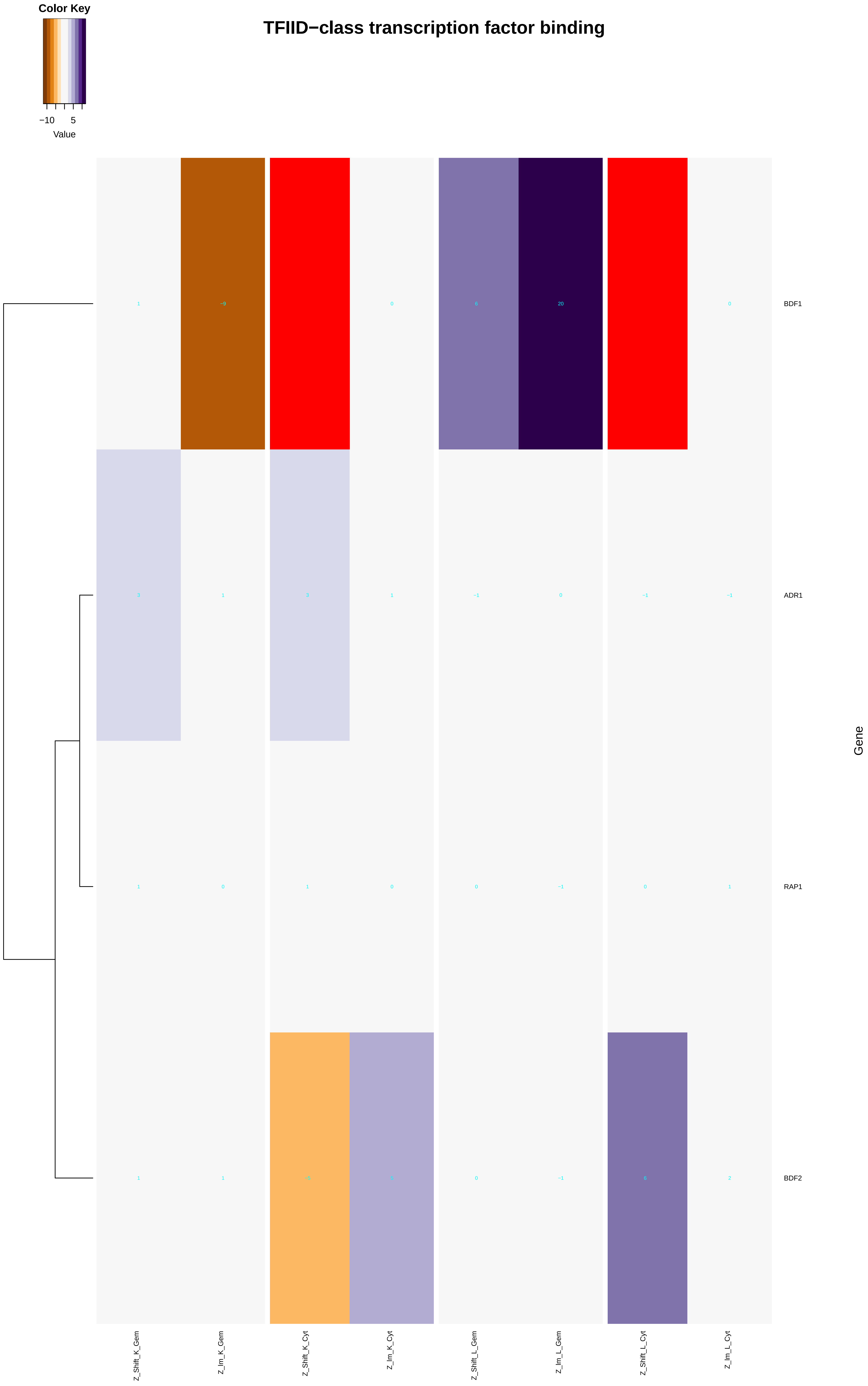

Color Key
−10
5
Value
TFIID−class transcription factor binding
BDF1
ADR1
RAP1
BDF2
Gene
Z_Shift_K_Gem
Z_Im_K_Gem
Z_Shift_K_Cyt
Z_Im_K_Cyt
Z_Shift_L_Gem
Z_Im_L_Gem
Z_Shift_L_Cyt
Z_Im_L_Cyt

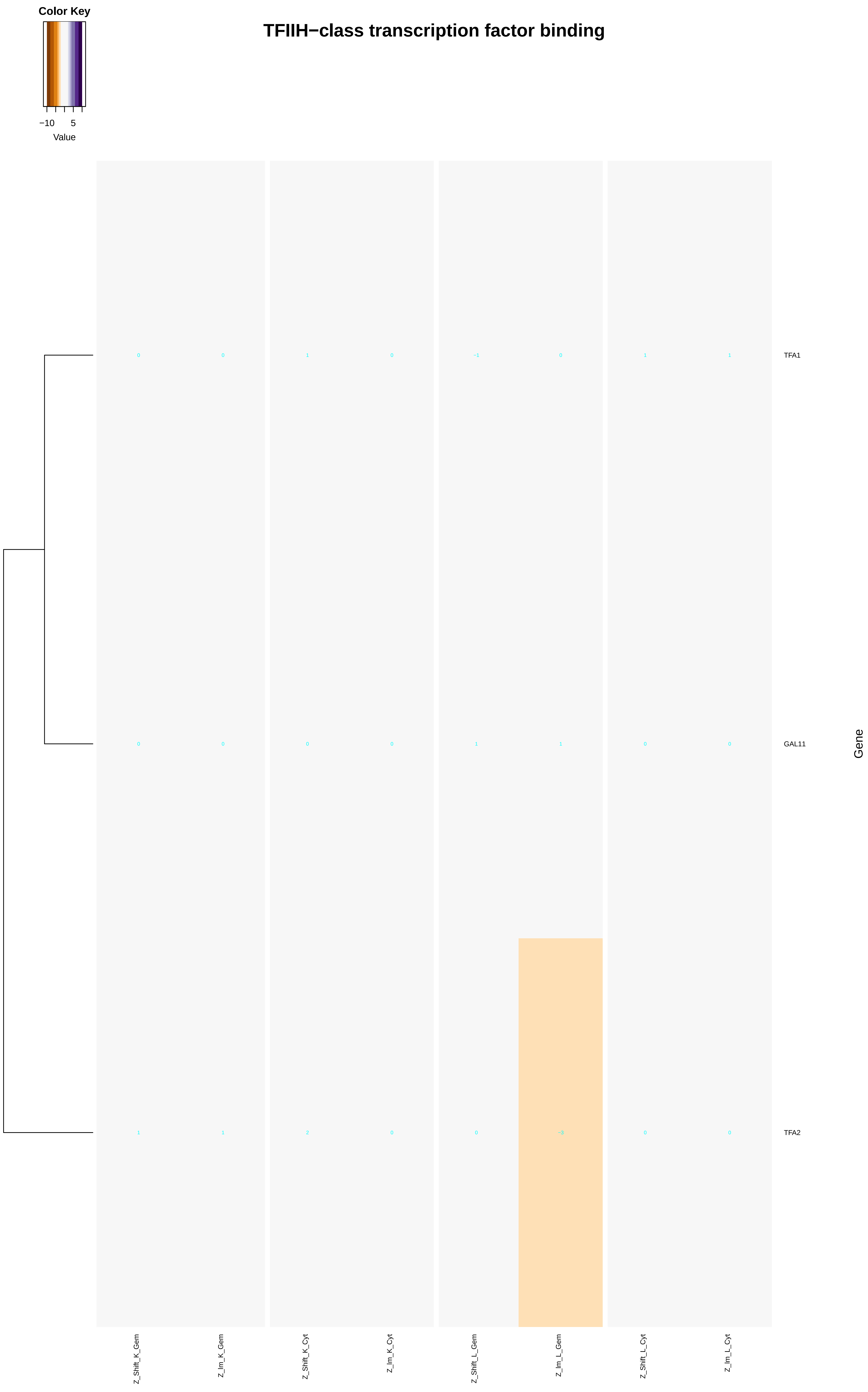

Color Key
−10
5
Value
TFIIH−class transcription factor binding
TFA1
GAL11
TFA2
Gene
Z_Shift_K_Gem
Z_Im_K_Gem
Z_Shift_K_Cyt
Z_Im_K_Cyt
Z_Shift_L_Gem
Z_Im_L_Gem
Z_Shift_L_Cyt
Z_Im_L_Cyt
